# Effects of 1,3-Butadiene, Isoprene, and Their Photochemical Degradation Products on Human Lung Cells

**DOI:** 10.1289/ehp.7022

**Published:** 2004-08-16

**Authors:** Melanie Doyle, Kenneth G. Sexton, Harvey Jeffries, Kevin Bridge, Ilona Jaspers

**Affiliations:** ^1^Environmental Sciences and Engineering, and; ^2^Center for Environmental Medicine, Asthma, and Lung Biology, University of North Carolina, Chapel Hill, North Carolina, USA

**Keywords:** 1,3-butadiene, air pollution, atmospheric chemistry, hydrocarbons, *in vitro*, interleukin-8, irradiative chambers, isoprene, lung epithelial cells, photochemical products

## Abstract

Because of potential exposure both in the workplace and from ambient air, the known carcinogen 1,3-butadiene (BD) is considered a priority hazardous air pollutant. BD and its 2-methyl analog, isoprene (ISO), are chemically similar but have very different toxicities, with ISO showing no significant carcinogenesis. Once released into the atmosphere, reactions with species induced by sunlight and nitrogen oxides convert BD and ISO into several photochemical reaction products. In this study, we determined the relative toxicity and inflammatory gene expression induced by exposure of A549 cells to BD, ISO, and their photochemical degradation products in the presence of nitric oxide. Gas chromatography and mass spectrometry analyses indicate the initial and major photochemical products produced during these experiments for BD are acrolein, acetaldehyde, and formaldehyde, and products for ISO are methacrolein, methyl vinyl ketone, and formaldehyde; both formed < 200 ppb of ozone. After exposure the cells were examined for cytotoxicity and interleukin-8 (*IL-8*) gene expression, as a marker for inflammation. These results indicate that although BD and ISO alone caused similar cytotoxicity and IL-8 responses compared with the air control, their photochemical products significantly enhanced cytotoxicity and *IL-8* gene expression. This suggests that once ISO and BD are released into the environment, reactions occurring in the atmosphere transform these hydrocarbons into products that induce potentially greater adverse health effects than the emitted hydrocarbons by themselves. In addition, the data suggest that based on the carbon concentration or per carbon basis, biogenic ISO transforms into products with proinflammatory potential similar to that of BD products.

The interest in 1,3-butadiene (BD) is not a new topic of concern within the environmental community. The Clean Air Act Amendments of 1990 (1990) added BD to the U.S. Environmental Protection Agency (EPA) hazardous air pollutants list. It ranks 36th among the top 50 most produced chemicals within the United States [Occupational Safety and Health Administration (OSHA) 2002] and is one of the top 33 in the Toxic Release Inventory (U.S. EPA 2001). Although the only natural form of BD known is from biomass combustion during natural forest fires, it has been used in production on a large scale since the 1930s [International Agency for Research on Cancer (IARC) 1992]. Although BD is used mainly in polymer manufacturing, it can also be found in large quantities in vehicle exhaust emissions, cooking oils, fungicides (captan and captafol), and cigarette smoke (Hughes et al. 2001; IARC 1992; Sorsa et al. 1996; Thorton-Manning et al. 1997). Although reported emissions fluctuate and can reach up to 3,000 tons/year within the United States (IARC 1992), outdoor concentrations typically range as low as 1–10 ppb in rural areas, with higher peaks closer to emitting industrial facilities (Hughes et al. 2001; IARC 1992).

Isoprene (ISO), like BD, is also emitted both naturally and anthropogenically into the environment. Biologic production of ISO is driven by photosynthesis and depends on both temperature and solar radiation (Guenther et al. 1993). ISO is the most predominant hydrocarbon emitted by a number of deciduous forest species (IARC 1994). It is also released into the air from rubber, thermoplastic and polymer industries, and cigarette smoke and is produced endogenously within the human body, being exhaled during respiration [National Toxicology Program (NTP) 2002]. Worldwide, ISO global emissions from vegetation range from 175 to 503 tons/year (Guenther et al. 1993), compared with the much larger estimated BD emissions in the United States alone. Similar to BD, outdoor concentrations of ISO range from 1 to 21 ppb but are generally < 10 ppb (IARC 1994; NTP 2002; Reimann et al. 2000). Although outdoor concentrations do not seem large enough to initiate immediate concern, indoor concentrations can range 5–10 times that of outdoor concentrations because of smoke from cigarettes, which emit both ISO and BD at about 3,100 and 400 μg/cigarette, respectively, into the air (NTP 2002).

Both ISO and BD react in the atmosphere through partially known chemical mechanisms, such as reactions with hydroxyl and other radicals as well as ozone, which are all created during photochemical processes. These reactions result in the formation of chemically specified and unspecified products, including those products that are not yet fully chemically identified or quantified (Atkinson and Arey 2003; Claeys et al. 2004; Feltham et al. 2000; Geiger et al. 2003; Jenkin et al. 1998; Larsen et al. 2001). Some of the unspecified products include multi-functional carbonyls such as hydroxyl carbonyls, dicarbonyls, and hydroxyl dicarbonyls (Chien et al. 1997; Liu et al. 1999; Tuazon and Atkinson 1990; Yu et al. 1995). Known ISO photochemical degradation products include methacrolein, methyl vinyl ketone, formaldehyde, 3-methylfuran, acetaldehyde, carbon monoxide, O_3_, organic nitrates, isoprene monoxide, peroxyacetyl nitrate (PAN), glycolaldehyde, hydroxyacetone, glyoxal and methylglyoxal, biacetyl, and Criegee biradicals (Atkinson and Arey 2003; Carter 1996; Palen et al. 1992; Sauer et al. 1999; Tuazon and Atkinson 1990; Yu et al. 1995). Known BD photochemical degradation products include acrolein, formaldehyde, organic nitrates, butadiene monoxide, CO, carbon dioxide, O_3_, PAN, 1,2-epoxy-3-butene, glycoladehyde, glycidaldehyde, 3-hydroxypropionaldehyde, and malonaldehyde (Atkinson 1990; Atkinson et al. 1994; Liu et al. 1999; Wayne et al. 1991; Yu and Jeffries 1997; Yu et al. 1995). Many of these products, for example, O_3_ and formaldehyde, are not merely formed through reactions with ISO and BD, and are generated during other photochemical transformation processes.

In this study, A549 cells were exposed to ISO, BD, or their photochemical degradation products. A549 cells are a model of respiratory epithelial cells with some type II-like cell characteristics that have been extensively used to assess the toxicity of air pollutants. Both ISO and BD alone are known sensory irritants with observable effects to the eyes, nasal passages, throat, and lungs (Alarie et al. 1998; U.S. EPA 2003; Wilkins et al. 2001). Many of the photochemical products for both ISO and BD have known health effects that have been studied in animals, humans, or both. For ISO, according to Rohr et al. (2002), the inflammatory effects from the products generated by reacting ISO and O_3_ were greater than the effects of either O_3_ or ISO alone. Wilkins et al. (2001) observed ISO products formaldehyde, formic acid, acetic acid, methacrolein, and methyl vinyl ketone causing sensory irritations for mice. Methacrolein alone causes irritation of the upper respiratory tract, painful sensation in human nasal cavities, and sensitization of the trigeminal nerve endings (Larsen and Nielsen 2000). Methyl vinyl ketone is a direct-acting irritant that targets the upper respiratory tract causing nasal lesions (Cunningham et al. 2001). Acrolein is considered highly acutely toxic, is a known sensory and upper respiratory irritant, and causes changes in respiratory function such as decrease in respiratory rate, rhythm, and amplitude [Gomes and Meek 2002; World Health Organization (WHO) 1992]. Studies show that formaldehyde is a moderate sensory irritant with carcinogenic evidence from occupational exposures (Liteplo et al. 2002; WHO 1989). Exposures to acetaldehyde alone also caused irritation of the eyes and mucous membranes, reddening of the skin, pulmonary edema, headache, and sore throat in humans and degeneration changes in the respiratory epithelium, trachea, and larynx in rats and hamsters (WHO 1995). With respect to all of the products created during photochemical reactions, O_3_ has been studied the longest and most in-depth. O_3_ toxicity includes inflammatory responses in sensory nerves, morphologic injury with inactivation of alveolar macrophage secretory enzymes, epithelial cytotoxicity, changes in airway resistance and respiratory rate, epithelial permeability, bronchoactive challenges, and changes in other pulmonary functions (Krishna et al. 1997; Lippmann 1989; Mehlman and Borek 1987; NTP 1994; WHO 1978). O_3_ is also a known animal carcinogen causing lesions in both F344/N rats and B6C3F1 mice (NTP 1994). Although respiratory health effects induced by some of the photochemical degradation products of ISO and BD are known, the toxicity of the entire photochemical reaction product mixture as it would occur in the atmosphere is unknown. In this study, we interfaced human lung epithelial cells with a smog chamber, allowing us to assess the toxicity of ISO, BD, and their photochemical degradation products in parallel.

## Materials and Methods

### Smog chambers.

Environmental irradiation chambers (also called smog chambers) that use sunlight can be used to study systems of natural transformation chemistry of pollutants (Jeffries et al. 1976; Jeffries and Fowler 2000; Sexton et al. 2004). Dual 150,000-L University of North Carolina (UNC) outdoor smog chambers made of FEP Teflon film (Livingstone Coating Corporation, Charlotte, NC) were used as photochemical reactors during the experiments. The chambers are located in Chatham County, North Carolina. Descriptive information about the chambers has been previously published (Jeffries et al. 1976; Jeffries and Fowler 2000). These chambers are ideal for studying chemical systems that are part of the real photochemical phenomena that occur within the atmosphere because the FEP Teflon film allows ultraviolet and visible regions of sunlight to react with the chemical components in the chamber.

Each chamber experiment was given a unique identification name symbolizing the date the experiment was performed. For example, AU2703 is an experiment performed on 27 August 2003. In addition, both chambers of the dual-chamber system were used during each experiment to expose two sets of cells simultaneously to different chemical mixtures, either reacted or unreacted with natural sunlight, using the same environmental conditions.

**Figure 1:**
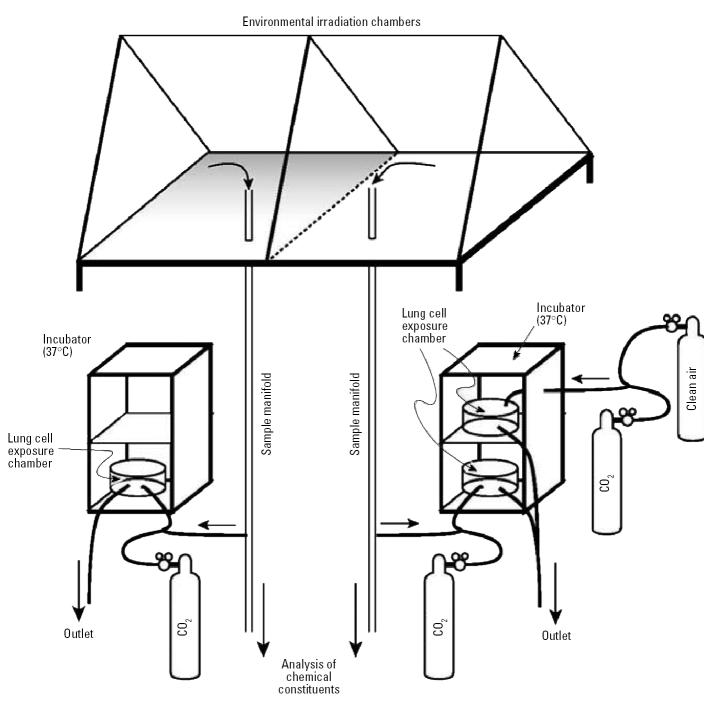
Schematic for the smog chamber–lung cell exposure setup.

For the experiments presented here, we used two injection protocols. For the first protocol, hydrocarbon (ISO or BD) and nitric oxide mixtures were either injected into a chamber and allowed to react with the sunlight or injected into a chamber after sundown. This protocol permitted the cells to be exposed either to photochemically generated reaction products or to the unreacted hydrocarbon and NO mixture. For these exposures, one chamber included a photochemically active system with 50 ppb NO and 200 ppb by volume (ppbV) of ISO or BD, whereas the other chamber contained the initial amount of hydrocarbon and NO but was kept in the dark (without sunlight) and therefore unreacted. These experiments were performed on different days with slightly different environmental conditions, including temperature, amount of sunlight, and humidity. In the second experimental protocol, one chamber was operated with 200 ppbV ISO and 50 ppb NO, whereas the other chamber had 200 ppbV BD and 50 ppb NO. This protocol directly compared the effects of photochemical products generated with ISO or BD under the exact same environmental conditions (sunlight, humidity, and photochemical reaction time). To prevent condensation inside the chamber after sundown, we used a chamber dehumidification system before the photochemical experiment to lower the dew point below the expected low temperature. Condensation would cause product loss because of adsorption into the moisture on the walls. In each experiment, a very small amount of carbon tetrachloride (CCl_4_), used as a tracer, was injected and then monitored to calculate the dilution within each chamber. During the first experimental protocol comparing ISO or BD and their photochemical products, the initial injections of 200 ppbV ISO (99%; Sigma-Aldrich, St. Louis, MO) or 200 ppbV BD (National Specialty Gases, Durham, NC) and 50 ppb NO were injected into one chamber at 4 hr before sunset (1500–1600 hr eastern daylight time) and allowed to react in the remaining sunlight. At sundown, these same initial amounts were injected into the opposite chamber side. During the second experimental protocol comparing the photochemical reaction products of ISO and BD, both 200 ppbV of ISO and BD and 50 ppb NO were added simultaneously into opposite sides of the chambers 4 hr before sunset and allowed to react with the remaining sunlight. The exposure to the lung cells began after sundown when photochemical reactions were terminated from the absence of sunlight. This short photochemical experimental design, conducted at the end of the day, stops the photo-oxidation sequence where the first-generation primary products are at their maximum concentration. Cells were exposed to the gaseous mixtures for 5 hr (Figure 1). After the exposures, all sets of cells were kept in the exposure chamber with control air plus 5% CO_2_ until transported to the laboratory.

### Cell culture and in vitro exposure.

A549 cells, a human lung epithelial cell line that has retained several alveolar type II-like cell characteristics, were used throughout this study. A549 cells were grown on membranous support (Costar-Clear Transwell inserts; Costar, Cambridge, MA) as described by Jaspers et al. (1997) in complete medium (F12K, 10% fetal bovine serum, antibiotics; all from Invitrogen, Carlsbad, CA). Upon confluency, the medium was exchanged for serum-free medium [F12K, 1.5 μg/mL bovine serum albumin (BSA), antibiotics; all from Invitrogen] several hours before exposure. Just before transport to the smog chamber site, media located in the apical chamber were aspirated, whereas media in the basolateral compartment remained. This facilitates direct exposure of lung epithelial cells to gaseous pollutants without significant interface of media while the cells are maintained with nutrients from the basolateral side. Triplicate sets of A549 cells were separately exposed to ISO plus nitrogen oxides (NO_x_), BD plus NO_x_, photochemical degradation products of ISO plus NO_x_, or photochemical degradation products of BD plus NO_x_ for 5 hr. Nine hours after exposure, cells were examined for cytotoxicity and interleukin-8 (*IL-8*) gene expression, as an indicator of inflammatory responses.

Primary human bronchial cells were obtained from healthy nonsmoking adult volunteers by cytologic brushing at bronchoscopy after they provided informed consent. The protocols for the acquisition of the primary human bronchial epithelial cells were reviewed and approved by the University of North Carolina Institutional Review Board. Primary human bronchial epithelial cells were expanded to passage 2 in bronchial epithelial growth medium (Cambrex Bioscience Walkersville, Inc., Walkersville, MD) and then plated on collagen-coated filter supports with a 0.4 μM pore size (Trans-CLR; Costar) and cultured in a 1:1 mixture of bronchial epithelial cell basic medium and Dulbecco modified Eagle medium:high glucose with l-glutamine with SingleQuot supplements (Cambrex Bioscience Walkersville), bovine pituitary extracts (13 mg/mL), BSA (1.5 μg/mL), and nystatin (20 U). Upon confluency, all-*trans*-retinoic acid was added to the medium, and air–liquid interface (ALI) culture conditions (removal of the apical medium) were created to promote differentiation. Mucociliary differentiation was achieved 18–21 days post-ALI.

### Smog chamber–lung cell exposure system.

The schematic shown in Figure 1 illustrates how the smog chambers were coupled to the *in vitro* exposure system. Inside tissue culture incubators we placed 8-L modular, cell-exposure chambers (MIC-10, Billups-Rothenberg, Del Mar, CA) that hold the tissue culture plates. Humidification of the exposure chamber was achieved by placing a dish of sterile water inside the chamber. The 8-L cell-exposure chambers have an inlet and an outlet connection for flowing gas through the exposure chamber. Sample lines directly coupled to the smog chambers through two externally circulated sample manifolds were used to provide chamber gases to the cells during exposure (Sexton et al. 2004). Three cell-exposure chambers were used throughout these studies, two of which were supplied with the gas mixtures from the smog chambers and one of which was supplied with medical-grade clean air. For each experiment, we exposed one set of A549 cells to clean air to control for potential variations induced by tissue culture or transport of the cells. In addition, the clean air control cell-exposure chamber was used to hold the cells during preexposure and postexposure periods. The cell-exposure chambers were ventilated with either humidified medical-grade air from a cylinder or with chamber air, both of which were mixed with 5% CO_2_. The addition of CO_2_ to chamber air was achieved using small pumps on the exhaust side and mass flow controllers (AALBORG, Orangeburg, NY). Humidification of the control cell exposure chamber was achieved by passing medical-grade air from the gas cylinder through two midget impingers in series (Ace Glass, Vineland, NJ), containing 15 mL HPLC-grade water (Fisher Scientific, Fairlawn, NJ). The impingers were heated at 28°C, which resulted in 50% relative humidity to match that in the smog chambers.

### Chemical analysis.

During each experiment, we used five chromatographic methods to monitor volatile organic compounds within the chambers. We used a Carle gas chromatograph (GC; Chandler Engineering, Tulsa, OK) to measure total hydrocarbon, which we used for assuring low background concentrations and measuring the initial injections. Samples were taken continually throughout the experiment, once each hour from each chamber, and analyzed with two Carle GCs using packed isothermal columns coupled to flame ionization detectors (FIDs). We used a Varian 3700 GC (Varian Inc. Scientific Instruments, Cary, NC) with electron capture detector, to measure CCl_4_ (our dilution tracer), PAN, and other N- or O-containing compounds; this GC was also used continually every 30 min throughout the duration of the experiment. We used a Varian 3400 capillary GC-FID with a Varian Saturn 2000 ion trap mass spectrometer (MS) to analyze air samples taken before, at the beginning, and during each exposure to help analyze for both known and unknown products created during the photochemical reactions. Formaldehyde was measured continuously, using the automated Dasgupta-diffusion-tube sampler to obtain aqueous formaldehyde, which is then mixed with buffered 2,4-pentanedione and measured with fluorescence (Dasgupta et al. 1988). O_3_ was measured using a U.S. EPA standard reference method based on photometry with a Thermo Environmental Instruments monitor (model 49; Thermo Environmental Instruments Inc., Franklin, MA). We measured NO_x_ using a U.S. EPA standard reference method based on chemiluminescence with a Monitor Labs monitor (model 98-41; Teledyne Monitor Labs Inc., Englewood, CO).

### Analysis of cytotoxicity and IL-8 expression.

Approximately 9 hr after exposure, basolateral supernatants from the exposed cells were collected and stored at –80°C until analysis for cytotoxicity and IL-8 expression. To determine cytotoxicity, the basolateral supernatants were analyzed for the release of cell lactate dehydrogenase (LDH) using a coupled enzymatic assay (Promega, Madison, WI), following the manufacturer’s instructions. Cytotoxicity was expressed as fold increase in LDH levels over the individual clean air control sample.

Total RNA was isolated using Trizol (Invitrogen) following the manufacturer’s instructions and analyzed for IL-8 mRNA levels by real-time reverse transcription polymerase chain reaction (RT-PCR) as described previously (Jaspers et al. 2001).

Basolateral supernatants were analyzed for IL-8 protein levels by ELISA (R&D Systems, Minneapolis, MN), following the manufacturer’s instructions. IL-8 protein levels were adjusted to account for the differences in viable cells that could produce and release IL-8 into the supernatant and expressed as fold increase over the individual clean air control sample.

### Statistical analysis.

The Student’s *t*-test was performed to compare the means of each set of results. To use this test, we made three assumptions: *a*) both sets of values have approximately normal distributions; *b*) they have roughly the similar variances; and *c*) each of the samples produced independent results. Data are presented as mean ± SEM, and a *p*-value < 0.05 was considered to be significant.

## Results

**Figure 2:**
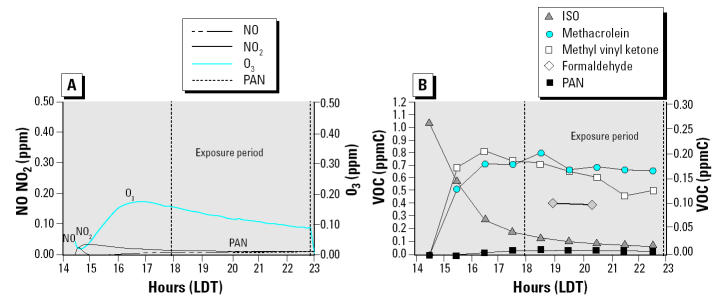
Representative time-series concentration plots for ISO photochemical reaction products for the experiment performed 13 October 2003. Abbreviations: LDT, local daylight time; ppmC, parts per million carbon; VOC, volatile organic compound. (*A*) Time course of NO_2_ and O_3_ formation after photochemical reaction of ISO and NO_x_. (*B*) Decay of ISO and production of methacrolein, methyl vinyl ketone, formaldehyde, and PAN (the concentration for ISO is given on the left *y*-axis, and the concentration for the other compounds is given on the right *y*-axis). The 200 ppbV ISO and 50 ppb NO underwent photochemical reactions from 4 hr before sunset until sundown, producing primarily first-generation photochemical degradation products. The dashed lines represent the 5-hr period the cells were exposed to the chamber contents.

**Figure 3:**
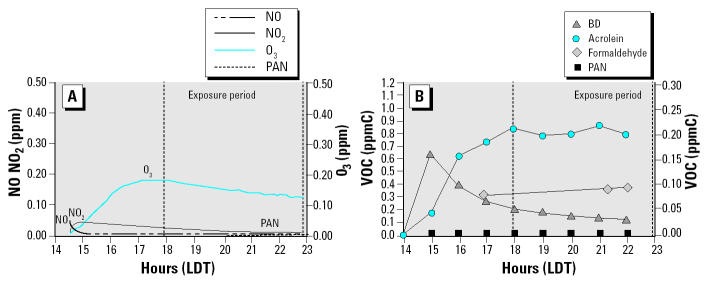
Representative time-series concentration plots for BD photochemical reaction products for the experiment performed 13 October 2003. Abbreviations: LDT, local daylight time; ppmC, parts per million carbon; VOC, volatile organic compound. (*A*) Time course of NO_2_ and O_3_ formation after photochemical reaction of ISO and NO_x_. (*B*) Decay of BD, and production of acrolein, formaldehyde and PAN (the concentration for BD is given on the left *y*-axis, and the concentration for the other compounds is given on the right *y*-axis). The 200 ppbV BD and 50 ppb NO underwent photochemical reactions from 4 hr before sunset until sundown, producing primarily first-generation photochemical degradation products. The dashed lines represent the 5-hr period the cells were exposed to the chamber contents.

The products generated during photochemical transformations of ISO or BD were identified by GC and confirmed using GC/MS. Table 1 summarizes the average levels and maximum concentrations of the known photochemical products derived from ISO or BD during the cellular exposure period on the indicated days. During the ISO experiments, the cells were exposed to practically the same concentrations of the first-generation products, primarily methacrolein, methyl vinyl ketone, and formaldehyde (within 0.030 ppm). The BD photochemical product concentrations, primarily acrolein, within the chamber during the exposure were also very similar (within 0.020 ppm). Therefore, each set of toxicologic results from the experiments performed on different days, but with the same hydrocarbon mixture, is comparable because of similar product concentrations generated and available during the exposure period. Figures 2 and 3 illustrate the photochemical smog chemistry within the two sides of the chamber during the experiment directly comparing photochemical reaction products formed with ISO or BD. These time-series concentration plots show the concentrations of the organic and inorganic species found within each chamber throughout the experiment. The formation and degradation of the inorganic species are shown in Figures 2A and 3A. After the initial NO injection, nitrogen dioxide, PAN, and O_3_ are generated from the reactions between the hydroxyl radicals and the initial injections of the ISO or BD and NO. Figures 2B and 3B show the photochemical degradation of the initial ISO or BD with the formation of the first-generation photochemical products from the time of injection through the end of the exposure period. Figures 2 and 3 show that the cell exposure to the chamber mixtures occurred directly after peak concentrations of the first-generation products were generated. The products generated during photochemical transformations of ISO or BD were identified by GC and confirmed using GC/MS. Data shown in Figures 2 and 3 and Table 1 indicate that, although photochemical reactions using ISO or BD as hydrocarbon precursors generate some of the same products (e.g., O_3_ or formaldehyde), several products are specific for either ISO or BD. In addition, the levels of formaldehyde and O_3_ produced by photochemical reactions with ISO or BD are different even though the initial carbon concentration reacted within the chamber was the same. For example, the O_3_ levels generated by photochemical reactions with ISO and exposed to the cells ranged from 0.118 to 0.130 ppm, whereas the O_3_ levels generated by photochemical reactions with BD ranged from 0.146 to 0.178 ppm.

**Figure 4:**
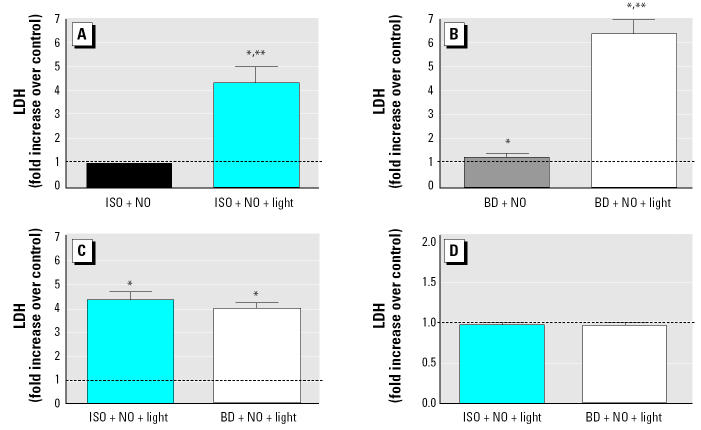
Analysis of cytotoxicity induced by exposure to NO (50 ppb) and ISO (200 ppbV), BD (200 ppbV), or their photochemical degradation products and expressed as fold increase of LDH over the control (mean ± SEM). (*A*) ISO without and with photochemical reaction. (*B*) BD without and with photochemical reaction. (*C*) ISO in one chamber and BD in the other chamber with photochemical reaction. (*D*) ISO in one chamber and BD in the other chamber with photochemical reaction. A549 cells were used in (*A*, *B*, and *C*) and differentiated human bronchial cells were used in (*D*); in all experiments, cells were exposed to mixtures for 5 hr. After exposure, supernatants were collected and evaluated for cytotoxicity (LDH release). See “Materials and Methods” for details of experiments. The dashed line indicates the normalized control value. *Significantly different from the control (*p* < 0.05). **Significantly different from the other side of the chamber (*p* < 0.05).

To determine whether the products generated by photochemical reactions with ISO or BD affect cell viability, we analyzed relative cytotoxicity induced by exposure to ISO, BD, or their photochemical product mixtures approximately 9 hr postexposure. Results from experiments performed on different days were combined, and cytotoxicity induced by ISO, BD, or their photochemical reaction products were expressed as fold increase over the respective control exposure to clean air. Figure 4A shows that ISO photochemical products induce a significant increase in cytotoxicity compared with ISO plus NO alone. Similarly, BD photochemical products are significantly more cytotoxic than BD plus NO alone, as shown in Figure 4B. Furthermore, directly comparing the cytotoxicity induced by photochemical products generated with ISO or BD, as shown in Figure 4C, suggests that ISO photochemical products have effects on cell viability similar to those of BD photo-chemical products. The results from the repeated ISO versus BD photochemical experiment using differentiated human bronchial cells derived from multiple individuals (Figure 4D) show that both ISO and BD photochemical products induced no significant change in cytotoxicity compared with the clean air exposure.

**Figure 5:**
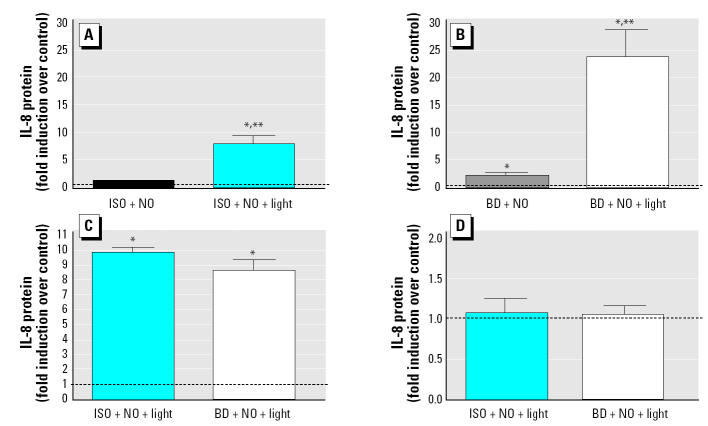
Analysis of IL-8 protein induced by exposure to NO (50 ppb) and ISO (200 ppbV), BD (200 ppbV), or their photochemical degradation products and expressed as fold increase over the control (mean ± SEM). (*A*) ISO without and with photochemical reaction. (*B*) BD without and with photochemical reaction. (*C*) ISO in one chamber and BD in the other chamber with photochemical reaction. (*D*) ISO in one chamber and BD in the other chamber with photochemical reaction. A549 cells were used in (*A*, *B*, and *C*) and differentiated human bronchial cells were used in (*D*); cells in all experiments were exposed to these mixtures for 5 hr. After exposure, supernatants were collected from the basolateral chambers and analyzed for IL-8 protein levels. See “Materials and Methods” for details of experiments. The dashed line indicates the normalized control value. *Significantly different from the control (*p* < 0.05). **Significantly different from the other side of the chamber (*p* < 0.05).

To examine whether photochemical reactions alter proinflammatory potential of ISO or BD, we compared the effects of ISO, BD, or their photochemical reaction products on IL-8 expression in both A549 and differentiated human bronchial cells. Figure 5A shows that ISO photochemical products induced a greater change in IL-8 expression (IL-8 protein induced) compared with clean air control. In contrast, ISO plus NO alone had no significant effect on IL-8 expression. Figure 5B demonstrates that BD photochemical products induced a significantly greater IL-8 expression compared with BD plus NO alone, which also enhanced IL-8 expression compared with the clean air control. Directly comparing the effects of photochemical products using A549 cells from either ISO or BD on IL-8 expression suggests that the products generated from ISO have a greater effect on IL-8 expression than do BD photochemical products (Figure 5C), although these data were not statistically significant (*p* = 0.06). The same experimental protocol was used to expose differentiated human bronchial cells to both ISO and BD photochemical degradation products. No significant changes in IL-8 release were seen (Figure 5D) in cells exposed to the photochemical mixtures compared with the air-exposed control cells.

**Figure 6:**
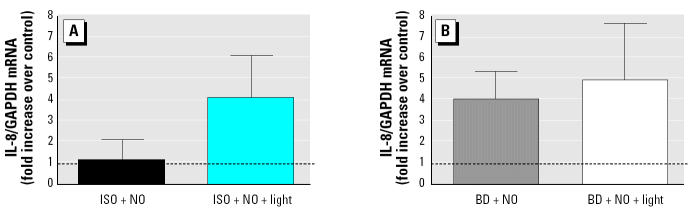
Analysis of IL-8 mRNA levels released by exposure to NO (50 ppb) and ISO (200 ppbV), BD (200 ppbV), or their photochemical degradation products in A549 cells and expressed as mean fold increase over the control (mean ± SEM). GADPH, glyceraldehyde-3-phosphate dehydrogenase. (*A*) ISO without and with photochemical reaction. (*B*) BD without and with photochemical reaction. A549 cells were exposed to these mixtures for 5 hr; after exposure, total RNA was isolated and analyzed for IL-8 mRNA levels by real-time RT-PCR. See “Materials and Methods” for details of experiments. The dashed line indicates the normalized control value.

We also measured IL-8 mRNA levels in experiments performed with A549 cells, as shown in Figure 6A and B. Similar to IL-8 protein levels released into the basolateral supernatants, IL-8 mRNA levels were enhanced by both ISO and BD photochemical products, although the levels did not reach statistical significance.

## Discussion and Conclusions

Previous research has shown that many photochemical products of ISO and BD are known sensory irritants to either animals or humans. However, the toxicities of these photochemical product mixtures produced in irradiative smog chambers using realistic atmospheric chemistry have not been previously studied. The advantage of using this approach, the smog chamber–cell exposure interface, to produce the photochemical mixtures is that all of the photochemical products are generated in the proper relative ratio, including the unspecified products. In this study, we examined cytotoxicity and *IL-8* gene expression induced by these photochemical gaseous mixtures using A549 cells, a human alveolar type II-like cell line, and differentiated human bronchial cells.

The data presented here demonstrate that ISO and BD photochemical products increased LDH release as well as IL-8 expression compared with their clean air controls. The IL-8 mRNA and protein data indicate that both biogenic ISO and anthropogenic BD, as they react within the atmosphere, generate products that are more potent inducers of *IL-8* gene expression than the unreacted volatile organic compounds. Although exposure to ISO and BD photochemical products generated significant levels of cytotoxicity and IL-8 expression in A549 cells, no significant effects were observed in differentiated human bronchial epithelial cells. These data indicate that differentiated human bronchial epithelial cells are less sensitive to the products generated after photochemical transformation of ISO and BD than A549 cells. Differentiated human bronchial epithelial cells release and are covered by a thin layer of mucus (Gray et al. 1996), which serves as a protectant against xenobiotics and inhaled gases (Schlosser 1999). Interactions and partitioning of inhaled agents within the mucus layer covering the epithelium of the tracheal-bronchial region can prevent these agents from reaching the underlying cell layer to induce cellular responses (Medinsky and Bond 2001). Alveolar epithelial cells lack such a protective mucus layer and could therefore be inherently more sensitive to inhaled gaseous species. Hence, the differences observed here may represent regional differences in sensitivity within the respiratory epithelium. Another reason for the observed differences in responses to ISO and BD photochemical products in A549 cells and differentiated human bronchial epithelial cells is that A549 cells are an immortalized cell line, whereas differentiated human bronchial epithelial cells are primary cells with a finite life span. Previous studies have shown that exposure of primary human bronchial epithelial cells and a bronchial epithelial cell line to O_3_ induced greater effects in the epithelial cell line than the in primary bronchial epithelial cells (Samet et al. 1992).

One concern when analyzing the results of the photochemical degradation products for both ISO and BD was the production of O_3_ and its known toxic health effects. These experiments were designed to produce the smallest concentration of O_3_ while continuing to keep the desired primary products within realistic ambient exposure concentration ranges. Previous studies have shown that A549 epithelial cells exposed to O_3_ concentrations less than those produced in these studies did increase IL-8 production (Jaspers et al. 1997), which would suggest that O_3_ is the major photochemical product causing the increase in IL-8 production. However, during the side-by-side experiment of photochemical products of ISO and BD, the average concentration of O_3_ inside the chambers was 120 ppb for ISO and 140 ppb for BD. Hence, if IL-8 production was solely based on O_3_ concentrations, the photochemical products of BD would produce a greater IL-8 response than would those of ISO. Because this is not the case, these data indicate that O_3_ is not the sole inducer of IL-8 protein secretion, but that other photochemical products generated during the reactions of ISO or BD with sunlight are causing the increase in IL-8 production. In addition, this suggests that although O_3_ concentrations are a good indicator of the adverse health potential of photochemical smog, one has to examine the entire photochemical mixture to estimate its toxic effect on the exposed population.

Analyses by GC-FID and GC/MS of resulting photochemical ISO and BD atmospheric reaction products showed the formation of many known products. The main ISO first-generation photochemical products formed were methacrolein, methyl vinyl ketone, formaldehyde, and O_3_, whereas BD formed acrolein, formaldehyde, and O_3_. Both experiments produced trace amounts of acetaldehyde, CO, PAN, and their respective monoxides. The sample results from GC and GC/MS analysis have not been explored for unknown products. Thus, several other photochemical products, besides O_3_, could have significant effects on IL-8 production in human lung cells. Data derived from these experiments could suggest potential agents in both the ISO and BD experiments that enhanced IL-8 production. In addition, in this article we describe the analysis of only known photochemical products formed during the ISO and BD reactions. Extensive chemical mechanism studies have been reported in the literature for both pollutants (Atkinson 1990; Atkinson and Arey 2003; Atkinson et al. 1994; Carter 1996; Liu et al. 1999; Palen et al. 1992; Sauer et al. 1999; Tuazon and Atkinson 1990; Wayne et al. 1991; Yu and Jeffries 1997; Yu et al. 1995), but the complete carbon balances have yet to be identified. Some of the unspecified products include multifunctional carbonyls such as hydroxyl carbonyls, dicarbonyls, and hydroxyl dicarbonyls (Chien et al. 1997; Liu et al. 1999; Tuazon and Atkinson 1990; Yu et al. 1995). Besides the mechanistic history of both chemicals, other experimental limitations of both sample collection and exposure design did not allow for the lung cells to be exposed to more polar compounds, nor could these compounds be quantified. Ongoing changes in our experimental setup will allow for these more polar products to be studied in the future.

In this study, we evaluated the quantification of inflammatory potential for primary photochemical products. Within the atmosphere these primary products typically continue to react, forming secondary and tertiary products. For outdoor exposures, then, the results shown here emphasize, by design, the primary pollutants. These first-generation products are continuously being produced as the precursors are continuously being emitted. But these primary pollutants continue to react as they are formed, not only limiting their maximum concentration but also producing secondary and tertiary products, which themselves have toxicity characteristics. The overall toxicity of pollutants must allow inclusion of residual primary, secondary, and tertiary products formed. Forthcoming experiments will include the comparison of primary, secondary, and tertiary products for different toxicity end points.

In this study we have demonstrated the importance of considering photochemistry when making decisions for outdoor air quality guidelines. Currently, air quality models include a predictive but not complete ISO mechanism; that is, they do not include detailed chemistry of the products formed. The data presented here demonstrate that the first-generation ISO photochemical products are more toxic than ISO itself, which indicates the need to better understand and to include the chemical mechanisms of the photochemical products in air quality models. Some studies suggest that ISO oxidation can contribute significantly to the total O_3_ production rate during O_3_ episodes (Biesenthal et al. 1997). O_3_ and its known adverse effects are of great health concern when creating guidelines related to exposure and release of pollutants that have the potential to produce a large amount of O_3_ once released into the atmosphere. This indicates the importance of increasing the inclusion of detailed chemistry of chemicals known to be biogenic such as ISO in photochemical models used to investigate O_3_ formation and health concerns to rural and urban outdoor exposures. This would enable people within the risk assessment community to make more realistic exposure guidelines using all sources of relevant toxicity, including nonanthropogenic sources that were previously overlooked.

## Figures and Tables

**Table 1:** Chemical analysis of chamber constituents.

	ISO		MACR		MVK		ISO MON		BD		Acrolein		Acetaldehyde		Form		PAN		O_3_		NO		NO_2_	
Chamber side	Ave	Max	Ave	Max	Ave	Max	Ave	Max	Ave	Max	Ave	Max	Ave	Max	Ave	Max	Ave	Max	Ave	Max	Ave	Max	Ave	Max
AU2703 ISO+NO+light vs. ISO+NO																								
Light	0.076	1.138	0.201	0.209	0.176	0.228	0.002	0.024	0.0	0.0	0.0	0.0	0.030	0.037	0.161	0.177	0.011	0.011	0.130	0.155	0.0	0.0	0.026	0.030
Dark	0.870	1.054	0.032	0.052	0.005	0.032	0.001	0.005	0.0	0.0	0.0	0.0	0.023	0.034	0.018	NA	0.001	0.001	0.008	0.012	0.0	0.0	0.026	0.077
ST1003 BD+NO+light vs. BD+NO																								
Light	0.0	0.0	0.0	0.0	0.0	0.0	0.0	0.0	0.042	0.777	0.197	0.237	0.013	0.047	0.096	0.109	0.001	0.002	0.178	0.210	0.005	0.018	0.012	0.025
Dark	0.0	0.0	0.0	0.0	0.0	0.0	0.0	0.0	0.593	0.738	0.029	0.171	0.010	0.054	0.004	0.004	0.001	0.001	0.011	0.295	0.010	0.031	0.022	0.045
ST2403 BD+NO+light vs. BD+NO																								
Light	0.0	0.0	0.0	0.0	0.0	0.0	0.0	0.0	0.125	0.713	0.184	0.255	0.023	0.044	0.083	0.093	0.001	0.002	0.154	0.203	0.001	0.022	0.001	0.013
Dark	0.0	0.0	0.0	0.0	0.0	0.0	0.0	0.0	0.686	0.753	0.017	0.033	0.005	0.017	0.004	0.004	0.001	0.001	0.011	0.060	0.001	0.019	0.022	0.037
OC1303: ISO+NO+light vs. BD+NO+light																								
ISO	0.116	1.040	0.176	0.202	0.153	0.205	NA	NA	0.0	0.0	0.0	0.0	NA	NA	0.102	0.103	0.010	0.011	0.118	0.156	0.001	0.005	0.013	0.022
BD	0.0	0.0	0.0	0.0	0.0	0.0	0.0	0.0	0.170	0.625	0.197	0.206	NA	NA	0.087	0.092	0.001	0.002	0.146	0.177	0.001	0.007	0.012	0.024

Abbreviations: AU, August; Ave, average concentration of exposure over the 5-hr period; Form, formaldehyde; ISO MON, ISO monoxide; MACR, methacrolein; Max, maximum concentration produced during the experiment; MVK, methyl vinyl ketone; NA, not available; OC, October; ppmC, parts per million carbon; ST, September. Light indicates the side that was photochemically reacted during each experiment, and dark indicates the side that remained unreacted.
